# Hungry for an intervention? Adolescents’ ratings of acceptability of eating-related intervention strategies

**DOI:** 10.1186/s12889-015-2665-6

**Published:** 2016-01-05

**Authors:** F. Marijn Stok, Denise T. D. de Ridder, Emely de Vet, Liliya Nureeva, Aleksandra Luszczynska, Jane Wardle, Tania Gaspar, John B. F. de Wit

**Affiliations:** 1 Psychological Assessment and Health Psychology, University of Konstanz, PO Box 47, 78457 Konstanz, Germany; 2 Department of Clinical and Health Psychology, Utrecht University, Utrecht, The Netherlands; 3 Wageningen University and Research Centre, Communication, Philosophy and Technology, Centre for Integrative Development, Wageningen, The Netherlands; 4 Department of Business Administration, University of Aarhus, Aarhus, Denmark; 5 Psychology Department in Wroclaw, University of Social Sciences and Humanities, Warsaw, Poland; 6 Trauma, Health, & Hazards Center, University of Colorado, Boulder, CO USA; 7 Department of Epidemiology & Public Health, University College London, Health Behaviour Research Centre, London, UK; 8 Faculty of Human Kinetics, Technical University of Lisbon, Lisbon, Portugal; 9 National Centre in HIV Social Research, University of New South Wales, Sydney, Australia

**Keywords:** Overweight, Eating behavior, Adolescents, Prevention, Interventions, Public policy

## Abstract

**Background:**

Effective interventions promoting healthier eating behavior among adolescents are urgently needed. One factor that has been shown to impact effectiveness is whether the target population accepts the intervention. While previous research has assessed adults’ acceptance of eating-related interventions, research on the opinion of adolescents is lacking. The current study addressed this gap in the literature.

**Methods:**

Two thousand seven hundred sixty four adolescents (aged 10–17 years) from four European countries answered questions about individual characteristics (socio-demographics, anthropometrics, and average daily intake of healthy and unhealthy foods) and the acceptability of ten eating-related intervention strategies. These strategies varied in type (either promoting healthy eating or discouraging unhealthy eating), level of intrusiveness, setting (home, school, broader out-of-home environment), and change agent (parents, teacher, policy makers).

**Results:**

Based on adolescents’ acceptability ratings, strategies could be clustered into two categories, those promoting healthy eating and those discouraging unhealthy eating, with acceptability rated significantly higher for the former. Acceptability of intervention strategies was rated moderate on average, but higher among girls, younger, overweight and immigrant adolescents, and those reporting healthier eating. Polish and Portuguese adolescents were overall more accepting of strategies than UK and Dutch adolescents.

**Conclusions:**

Adolescents preferred intervention strategies that promote healthy eating over strategies that discourage unhealthy eating. Level of intrusiveness affected acceptability ratings for the latter type of strategies only. Various individual and behavioral characteristics were associated with acceptability. These findings provide practical guidance for the selection of acceptable intervention strategies to improve adolescents’ eating behavior.

## Background

The high prevalence of overweight and obesity in adolescence is a pressing public health issue, with rates of overweight ranging between 10 and 40 percent among European adolescents [1]. Childhood obesity has been identified by the World Health Organization [2] as one of the most serious public health challenges of the 21st century. Obesity is associated with the development of non-communicable diseases, including cardiovascular diseases and diabetes. The risk of developing these diseases increases with earlier age of onset and longer duration of obesity, increasing the vulnerability of those who develop excess weight before adulthood [3, 4]. Preventing and treating obesity in youngsters is a public health priority, and policy makers have designed and implemented many policies and interventions aimed at preventing and decreasing overweight and obesity in children and adolescents [5]. So far, however, these strategies have not been able to successfully reduce obesity rates [6, 7].

### Public support for diet-related interventions strategies

An important prerequisite for the successful implementation of interventions is that the target population accepts them [8–11]. This is important both on a political level, because politicians are unlikely to favor policies that may lose them votes, and in terms of potential effectiveness, because behavior change is unlikely to occur following implementation of strategies that the target population does not consider acceptable [8, 9, 11]. Research into popular opinion regarding health promotion policies in general, and diet-related policies in particular, has soared in the past decade. This research has consistently found that public support tends to be highest for the least intrusive policies, such as education programs and the promotion of healthy eating, and lowest for intrusive, restrictive policies, such as bans on advertising or selling unhealthy products, or taxation of unhealthy products [8–11].

A further consistent finding is that public support is generally higher for policies to improve the diet of children and adolescents [9, 10, 12], and remains high, albeit at a somewhat lower level [12], if these policies are restrictive or intrusive. However, these findings are exclusively based on research among adults, and may reflect a general tendency to be supportive of restrictive policies as long as they do not affect oneself [8].

### The adolescent perspective

An important open question is thus how youngsters themselves feel about potential intervention strategies aimed at improving their dietary behavior. While the political argument for the importance of policy acceptability is less applicable to adolescents, as they are not yet voters, the policy effectiveness argument may be especially relevant for adolescents. Adolescents are developing their own identity, opinions and ideas [13], and typically portray a stronger than average negative response to the sense of being pushed in a certain direction [14, 15]. Intervention strategies that give adolescents the feeling that their behavior is being steered or their freedom of choice is being restricted may induce feelings of resistance to the behavior proposed in the intervention [16]. This in turn may cause psychological reactance [17], which adolescents may attempt to resolve by acting out against the suggested behavior (i.e., doing exactly that what is being warned against), or by derogating the source of the message.

It is therefore critical to understand how adolescents themselves feel about various possible intervention strategies to improve healthy diets, which types of strategies they find acceptable and which not. In the current study, we investigate the extent to which adolescents accept ten possible strategies aimed at improving their eating behavior. Three research questions will be addressed: (1) To what extent do adolescents accept various intervention strategies; (2) can single strategies be clustered into meaningful categories; and (3) which individual and behavioral characteristics are associated with acceptance for these clusters of intervention strategies.

## Methods

### Respondents and procedure

This study was conducted as part of the “Temptations to Eat Moderated by Personal and Environmental Self-regulatory Tools” (TEMPEST) project undertaken in nine European countries. The presented data were collected through a larger self-report survey in four of these countries, investigating environmental influences on the self-regulation of children’s and adolescents’ eating behavior. Previous publications have reported on other instruments included in this survey, which are not discussed in the current paper [18–21]. The four countries in which data were collected are Poland, Portugal, the Netherlands and the United Kingdom (UK). These four countries were selected because they differ predictably with respect to socio-economic and socio-cultural characteristics, as well as regarding childhood overweight prevalence [1]. Respondents were recruited via primary schools and high schools (*N* = 24). Our aim was to recruit 600 participants per country, distributed across our targeted age range of 10 to 17 years. Schools were selected based on availability and willingness to participate in the study. However, to ensure a diverse sample, care was taken to select schools from both rural and urban areas (50.9 % versus 49.1 %, respectively), and from areas of both low and high socio-economic status (31.4 % versus 68.6 %, respectively). A total of 2764 adolescents between the ages of 10 and 17 years participated in the study.

In each country, an authorized review board granted ethical approval for the study or granted exemption from the requirement to seek approval. Specifically, in the UK, ethics approval was granted by the University College London Research Ethics Committee. In Poland, the International Review Board – KEBE of the University of Social Sciences and Humanities in Warsaw granted approval, and in Portugal approval was granted by the ethics committee of the São João Hospital Centre. In The Netherlands the Central Committee on Research Involving Human Subjects indicated that ethics approval did not have to be sought for this study. Consent (active or passive, depending on country regulations) for participation was sought from parents or caregivers. Specifically, active parental or caregiver consent was sought in Poland, while passive consent was sought in the UK and The Netherlands. In Portugal, internal school regulations differed on whether active or passive consent should be obtained, and both approaches were used in this country following each school’s guidelines. In all countries, assent was obtained from the respondents on the day of the study by informing the pupils that they were free to decide if they wanted to participate in the study or not. Respondents could discontinue participation at any time. Respondents filled out the questionnaire during class hours and in their regular class setting. Their teacher and a research assistant were present in the classroom during data collection.

### Measures

The questionnaire was prepared in English, translated into each country’s (main) language, and back-translated into English. Where required, translations were revised. In the current study, only the measures described below were included. The full questionnaire is available from the authors upon request. All items were self-reported.

Various individual and behavioral characteristics were assessed. Respondents reported their *age* and *gender*. They also self-reported their height and weight, from which *Body Mass Index (BMI)* was calculated and categorized as underweight, normal weight, overweight or obese based on cut-off points for age and gender described by the International Obesity Task Force [22]. For ease of analysis, a dichotomous *weight class* variable was calculated (normal weight or underweight vs. overweight or obese). *Immigrant status* (native vs. immigrant) was determined based on the language respondents reported speaking with their parents [23]. *Family affluence* was assessed with the child-appropriate Family Affluence Scale (FAS [24]). Using the procedure outlined by the authors of the scale, three affluence categories (low vs. medium vs. high) were created. Average daily intake of four types of food (pieces of fruit, servings of vegetables, number of unhealthy snacks and number of soft drinks) was assessed using a 5-point scale (ranging from 0 = zero per day to 4 = four or more per day). Two composite scores were created reflecting *healthy eating* (fruit and vegetables) and *unhealthy eating* (unhealthy snacks and soft drinks).

*Acceptability of intervention strategies to improve eating behavior:* a list of ten possible intervention strategies, aimed specifically at adolescent populations, was generated by the research team, inspired by previous research and inventories of available strategies [5–8]. Care was taken to vary the intervention strategies on four dimensions: type, intrusiveness, setting, and change agent. With regard to type of strategy, we selected an equal number of strategies aimed at promoting healthy eating behavior and at discouraging unhealthy eating behavior. With regard to intrusiveness, following the example of Diepeveen and colleagues [8], we used the Nuffield intervention ladder [25] to select strategies that varied in level of intrusiveness and limitation of freedom of choice. With regard to setting, we selected strategies occurring in various environmental contexts (e.g. home, school, broader out-of-home environment). Finally, regarding change agent, we varied the person delivering the intervention strategy (e.g. parents, teachers, policy makers). Acceptability of each strategy was assessed by asking respondents to indicate extent of agreement with use of the strategy (e.g. “Schools should not sell unhealthy snacks and soft drinks”; “Healthy foods and drinks should be cheaper than unhealthy products”; for all statements, see Table 2); responses were given on a 5-point Likert scale ranging from 1 (strongly disagree) to 5 (strongly agree).

### Data analysis

First, acceptability of each of the strategies and overall acceptability across all ten strategies were calculated. Second, a principal component analysis was conducted to determine whether the ten strategies could be grouped into higher-order categories, and to determine which dimension(s) (type, level of intrusiveness, setting, or change agent) this grouping would be based on. Third, a paired samples *t*-test was used to assess whether acceptability differed significantly between these higher-order categories. Third, regression analyses were conducted to determine whether individual and behavioral characteristics (i.e., socio-demographic and anthropometric characteristics, and average daily healthy and unhealthy food intake) were associated with acceptance for specific categories of interventions.

## Results

Descriptive statistics of the sample are depicted in Table 1. Respondents’ mean age was 13.2 years (*SD* = 1.9, range 10–17); 50.9 % were boys and 49.1 % were girls. Most respondents (94.2 %) spoke the country’s native language at home. Most indicated high family affluence (52.5 %), with fewer respondents from families of medium (35.8 %) and low (11.7 %) affluence. Using self-reported height and weight data, it was estimated that most respondents (71.5 %) had a BMI in the normal range. Of the remaining respondents, 11.9 % were underweight, 13.4 % were overweight and 3.2 % were obese. On average, respondents consumed 4.0 portions of fruit and vegetables per day (*SD* = 2.3), and 3.9 unhealthy snacks and soft drinks (*SD* = 2.4).

**Table 1:** Means and standard deviations or percentages for the main variables under study

Variable	Mean (or percentages)	Standard Deviation
Age	13.2 years (range 10–17)	1.9
Gender	50.9 % boys	n.a.
	49.1 % girls	
Weight status	11.9 % underweight	n.a.
	71.5 % normal weight	
	13.4 % overweight	
	3.2 % obese	
Family affluence status	11.7 % low FAS	n.a.
	35.8 % medium FAS	
	52.5 % high FAS	
Immigrant status	94.2 % native	n.a.
	5.8 % immigrant	
Overall acceptance of intervention strategies	3.20 (range 1–5)	0.83
Healthy food intake (average daily intake)	4.0 (range 0–10)	2.3
Unhealthy food intake (average daily intake)	3.9 (range 0–10)	2.4

### Support for interventions strategies

Across all ten strategies, mean endorsement was slightly above the mid-point of a five-point scale ranging from 1 to 5 (*M* = 3.20, *SD* = 0.83, see Table 1), corresponding to the response ‘neither agree nor disagree’. Table 2 provides the acceptability ratings of all ten strategies, ordered from lowest to highest. There was considerable variation in the extent to which respondents deemed the various strategies to be acceptable (see Table 2), with mean responses per strategy ranging from 2.28 (indicating mild unacceptability) to 3.82 (indicating mild acceptability) on a five-point scale. Adolescents were most accepting of the strategy of parents discussing the importance of healthy eating with their children (*M* = 3.82, *SD* = 1.07), and least accepting of the strategy of banning the sale of unhealthy foods and drinks to young people (*M* = 2.28, *SD* = 1.29).

**Table 2:** Intervention strategies, ranging from lowest to highest endorsement, with mean endorsement (*SD*), component loadings (loadings > .40 indicated in bold) from the Principal Component Analysis, and item-total correlations with the relevant subscale

Intervention strategy	Mean acceptability (SD)	PCA factor loadings component 1	PCA factor loadings component 2	Item-total correlations subscale “promoting healthy eating”^b^	Item-total correlations subscale “discouraging unhealthy eating”^b^
1. Unhealthy foods and drinks should be banned for sale to young people	2.28 (1.29)	.10	**.87**		.82
2. Advertising of snacks and soft drinks to young people should be prohibited	2.54 (1.25)	.16	**.82**		.81
3. The price of snacks and soft drinks should be increased so that young people consume less	2.78 (1.33)	.27	**.76**		.81
4. Schools should not sell unhealthy snacks and soft drinks	2.92 (1.27)	.36	**.69**		.78
5. Snacks and soft drinks should have health warning labels^a^	3.30 (1.25)	**.49**	**.49**		.69
6. It is a good idea to have rules at home about eating fruits and vegetables	3.55 (1.10)	**.80**	.21	.82	
7. Teachers should encourage young people to eat healthily	3.55 (1.10)	**.70**	.35	.78	
8. Young people should learn more about healthy eating in school	3.62 (1.10)	**.80**	.23	.83	
9. Healthy foods and drinks should be cheaper than unhealthy products	3.64 (1.18)	**.61**	.26	.70	
10. It is important that parents talk with their children about the importance of healthy eating	3.82 (1.07)	**.84**	.04	.79	
*Principal Component Analysis*	*Eigenvalue*	4.86	1.47		
	*Explained variance*	48.6 %	14.7 %		
	*Cronbach’s alpha*			.84	.84

^a^Item 5 loaded equally on both factors. It is included in component 2 based on theoretical reasoning. Therefore, the subscale “strategies promoting healthy eating” was composed of items 6 to 10, while the subscale “strategies discouraging unhealthy eating” was composed of items 1 to 5. ^b^All correlations significant at *p* < .01

### Factorial structure of acceptability of intervention strategies

To determine whether the strategies could be clustered into meaningful categories based on the acceptability ratings, a principal component analysis with orthogonal (varimax) rotation was performed. Bartlett’s test of sphericity (*Χ* [2] (45) = 11144.05, *p* < .001) indicated satisfactory correlations between items. Two components had eigenvalues >1 (Kaiser’s criterion). Inspection of the scree plot confirmed the adequacy of a two-factor solution, with the point of inflexion occurring at the third factor. Together these two factors explained 63.3 % of the variance. Table 2 shows the factor loadings after rotation. Inspection of the items clustering on each component indicated that component 1 (five items) pertained exclusively to strategies aimed at promoting healthy eating, and component 2 (five items) to strategies aimed at discouraging unhealthy eating. While item five loaded equally on both factors, it was included component 2 based on conceptual grounds, as it reflects a strategy aimed at discouraging unhealthy eating. A paired samples *t*-test showed that adolescents gave higher acceptability ratings for strategies promoting healthy eating (*M* = 3.63, *SD* = 0.87) than strategies discouraging unhealthy eating (*M* = 2.77, *SD* = 1.00), *t* (2620) = 50.31, *p* < .001, *d* = 0.92. The two subscales correlated moderately strongly (*r* = .57, *p* < .001).

### Factors associated with acceptability of the two categories of intervention strategies

Having established two clear categories of intervention strategies, we proceeded to investigate which individual and behavioral characteristics (age, gender, country, overweight status, family affluence, immigrant status, and healthy and unhealthy food intake) were associated with acceptability of the two categories of strategies. The results (see Table 3) showed that reported acceptability of strategies promoting healthy eating was higher among younger adolescents, girls, Polish and Portuguese adolescents, immigrant adolescents, adolescents eating more healthy food items, and adolescents eating fewer unhealthy food items. Acceptability of strategies promoting healthy eating was also marginally higher among overweight and obese adolescents. There was no association with family affluence. Acceptability of strategies discouraging unhealthy eating was higher among younger adolescents, overweight and obese adolescents, Polish and Portuguese adolescents, adolescents eating more healthy food items and adolescents eating fewer unhealthy food items. Acceptability of strategies discouraging unhealthy eating was also marginally higher among girls and immigrants. There was no association with family affluence. Additional analyses (data not shown) using each country as reference category in turn indicated that, across both categories of intervention strategies, Portuguese adolescents gave higher acceptance scores than Polish adolescents (although the difference for strategies promoting healthy eating was only marginally significant), and that Polish adolescents gave higher acceptance scores than Dutch and UK adolescents.

**Table 3:** Multiple regression analyses of acceptability of intervention strategy categories on individual and behavioral characteristics

	Acceptability of strategies promoting healthy eating^a^			Acceptability of strategies discouraging unhealthy eating^b^		
Individual and behavioral characteristics	*B* (*SE*)	*β*	*p*	*B* (*SE*)	*β*	*p*
Age	-.05 (.01)	-.12	<.001	-.09 (.01)	-.19	<.001
Gender (0 = boy, 1 = girl)	.17 (.04)	.10	<.001	.08 (.04)	.04	.055
Country: Netherlands vs. UK	.04 (.06)	.02	.524	-.01 (.07)	-.00	.919
Country: Poland vs. UK	.19 (.05)	.11	.001	.24 (.06)	.11	<.001
Country: Portugal vs. UK	.28 (.06)	.13	<.001	.40 (.07)	.16	<.001
Overweight status (0 = not overweight, 1 = overweight)	.10 (.05)	.04	.054	.25 (.06)	.09	<.001
Family affluence: high vs. low	.03 (.06)	.02	.604	.09 (.07)	.04	.192
Family affluence: medium vs. low	.03 (.06)	.02	.580	.10 (.07)	.05	.140
Immigrant status (0 = native, 1 = immigrant)	.22 (.08)	.06	.008	.16 (.10)	.04	.096
Healthy food intake index	.06 (.01)	.16	<.001	.05 (.01)	.11	<.001
Unhealthy food intake index	-.06 (.01)	-.17	<.001	-.07 (.01)	-.17	<.001

^a^R^2^ = .11, *F* (11,2029) = 23.70, *p* < .001; ^b^R^2^ = .14, *F* (11,2026) = 29.26, *p* < .001

## Discussion

This study investigated adolescents’ acceptance of intervention strategies aimed at improving their eating behavior. While previous studies have investigated the extent to which adults accept interventions targeted at adolescents, research into the extent to which adolescents themselves find such interventions acceptable is scarce. Overall, adolescents rated the strategies as neither acceptable nor unacceptable, but acceptability was higher among younger adolescents, girls, overweight or obese adolescents, immigrant adolescents and those with healthier average daily food intake (as evidenced by higher intake of healthy, and lower intake of unhealthy, foods). Portuguese adolescents were most accepting of the strategies, followed by Polish adolescents, while Dutch and UK adolescents reported the lowest acceptability. These findings partially align with previous findings from studies investigating acceptability of interventions among adult populations. For example, previous studies have also found that women tend to be more accepting of interventions [8, 9] and that those reporting unhealthier behaviors are less accepting of interventions [8, 26].

Novel findings are that younger adolescents, overweight and obese adolescents, Portuguese and Polish adolescents, and immigrant adolescents are more accepting of intervention strategies. Younger adolescents’ higher acceptance as compared to older adolescents may be due to the fact that the need for autonomy and personal agency increase during adolescence [27, 28], which may inversely relate to acceptance of intervention strategies imposing rules and regulations. There are at least two possible explanations for the finding that overweight and obese adolescents reported higher acceptability than normal weight adolescents. On the one hand, it could be that these adolescents experience a genuine need for outside help, reflected in higher acceptability. On the other hand, their higher acceptability ratings may also be an expression of social desirability. The finding that Portuguese and Polish adolescents reported higher acceptability than Dutch and UK adolescents may be due to the differences between these countries in individualism [29]. Corresponding to our finding that Portuguese adolescents are most accepting of the interventions strategies, Hofstede [29] reports that Portuguese culture is highly collectivist, more so than Polish culture and much more so than Dutch and UK culture (correspondingly, we found that Polish adolescents, while reporting lower acceptability of intervention strategies than their Portuguese peers, reported higher acceptability than Dutch and UK adolescents). A more individualistic culture may translate into less support for rules and regulations imposed by others, as in such a culture, people typically experience a higher need for personal agency and self-determination [29]. The same argument could apply to our finding regarding higher acceptability ratings by immigrant adolescents, as their native culture may be more collectivist than that of the host country.

### Factorial structure of the strategies

The strategies were found to be separable into two meaningful categories, based on the type of strategy: the first category reflected policies aimed at promoting healthy eating, while the second reflected policies aimed at discouraging unhealthy eating. The dimension of “type of strategy” was thus more influential for acceptability than the other dimensions (level of intrusiveness, setting and change agent). This is very clear, for example, with regard to the two items reflecting price manipulations, which only differ in type of strategy and share the same level of intrusiveness, the same setting and the same change agent. Crucially, the item reflecting an increase in the price of unhealthy foods (i.e., a strategy discouraging unhealthy eating) was rated as the second-lowest acceptable strategy, while the item reflecting a decrease in the price of healthy foods (i.e., a strategy promoting healthy eating) was rated as the second-highest acceptable strategy.

While type of strategy was thus clearly the most important dimension in terms of acceptability, the results suggest that the other dimensions also affected acceptability ratings, at least for strategies discouraging unhealthy eating. Regarding the level of intrusiveness, strategies discouraging unhealthy eating with lower levels of intrusiveness (according to the Nuffield intervention ladder [25]) were rated as more acceptable. Similar to adults [8, 10], adolescents thus show a preference for less intrusive strategies, at least where strategies aimed at discouraging unhealthy eating are concerned. Importantly, however, for strategies aimed at promoting healthy eating, adolescents did not show a preference for less intrusive interventions. This may point to an important implication, namely that adolescents actually accept intervention strategies, even when these are intrusive, as long as they are aimed at improving healthy eating behavior. Other studies have shown a similar preference of adolescents for promotion of the ‘right’ behavior over discouragement of the ‘wrong’ behavior [20].

In a similar vein, strategies discouraging unhealthy eating in a broader out-of-home setting and strategies delivered by policy makers as change agents were rated as less acceptable than strategies in a home or school setting and strategies delivered by parents or teachers. Again, for strategies aimed at promoting healthy eating, setting and change agent do not appear to have a systematic influence on acceptability, with scores varying across the different types of settings and change agents. It is important to note that the dimensions of setting and change agent are not completely separate dimensions but are intertwined to a certain extent (parents are more likely to be change agent for strategies in a home setting, for example). However, the distinction between the two is not redundant. For example, the strategy of prohibiting soft drinks and unhealthy schools refers to a school setting, but is delivered not by teachers but by policy makers.

### Limitations

The current study is not without limitations. An important limitation is that we assessed acceptability of only ten intervention strategies, which were determined by the authors. Many more strategies exist [30] which have not been included in the current analysis. Similar investigations should therefore be carried out for more systemically organized and exhaustive lists of intervention strategies, for example in order to determine whether the finding that adolescents indicate higher acceptability for less intrusive strategies aimed at discouraging unhealthy also holds across a larger array of strategies. Furthermore, it should be noted that one of our strategies (“Healthy foods and drinks should be cheaper than unhealthy products”) is not strictly a strategy, but rather the result of one or more strategies that could encompass either increasing the price of unhealthy food or decreasing the price of healthy food, or both. While our factor analysis indicates that our respondents primarily associated this item with the promotion of healthy eating, we have not directly tested this assumption.

Another limitation lies in the fact that we have currently only assessed associations of a limited number of individual characteristics (socio-demographics, BMI and average healthy and unhealthy food intake) with acceptability. While these characteristics have been found to be associated with policy support in earlier studies among adults [8, 10], several additional factors have been shown to be substantially associated with acceptance of interventions, such as attributions of the causes of obesity [30, 31], regulatory focus (promotion versus prevention [27]), and health risk concerns [26] or problem identification [10]. It would be important for future research on adolescents’ acceptance of eating-related interventions to take these additional factors into account.

## Conclusions

Adult support for intervention strategies aimed at improving youngsters’ eating behavior is typically high. However, less research has investigated the views of adolescents themselves with regard to such strategies. The present study aimed to contribute to filling this gap in the literature. The results demonstrated that adolescents are, overall, moderately accepting of such strategies. Importantly, level of acceptability was moderated by type of intervention strategy: adolescents reported higher acceptability for strategies aimed at increasing healthy eating behavior than for strategies aimed at decreasing unhealthy eating behavior. Furthermore, adolescents showed a preference for non-intrusive strategies over more intrusive ones, particularly with regard to strategies aimed at decreasing unhealthy eating. As it has been shown that intervention strategies are more likely to actually instigate behavior change when they are considered acceptable by the people they are targeted at [8, 11], these findings may hold important practical implications for policy makers and intervention planners.

## Availability of data and materials

Not applicable.
